# Radiation alters the cargo of exosomes released from squamous head and neck cancer cells to promote migration of recipient cells

**DOI:** 10.1038/s41598-017-12403-6

**Published:** 2017-09-29

**Authors:** Lisa Mutschelknaus, Omid Azimzadeh, Theresa Heider, Klaudia Winkler, Marcus Vetter, Rosemarie Kell, Soile Tapio, Juliane Merl-Pham, Stephan M. Huber, Lena Edalat, Vanja Radulović, Nataša Anastasov, Michael J. Atkinson, Simone Moertl

**Affiliations:** 1 Helmholtz Zentrum München, German Research Center for Environmental Health, Institute of Radiation Biology, Neuherberg, Germany; 2 Independent Scientist, Hofheimerstraße 6, Munich, Germany; 3 Helmholtz Zentrum München, German Research Center for Environmental Health, Research Unit Protein Science, München, Germany; 4 Department of Radiation Oncology, University of Tübingen, Tübingen, Germany; 5 Chair of Radiation Biology, Technical University of Munich, Munich, Germany

## Abstract

Radiation is a highly efficient therapy in squamous head and neck carcinoma (HNSCC) treatment. However, local recurrence and metastasis are common complications. Recent evidence shows that cancer-cell-derived exosomes modify tumour cell movement and metastasis. In this study, we link radiation-induced changes of exosomes to their ability to promote migration of recipient HNSCC cells. We demonstrate that exosomes isolated from irradiated donor cells boost the motility of the HNSCC cells BHY and FaDu. Molecular data identified enhanced AKT-signalling, manifested through increased phospho-mTOR, phospho-rpS6 and MMP2/9 protease activity, as underlying mechanism. AKT-inhibition blocked the pro-migratory action, suggesting AKT-signalling as key player in exosome-mediated migration. Proteomic analysis of exosomes isolated from irradiated and non-irradiated BHY donor cells identified 39 up- and 36 downregulated proteins. In line with the observed pro-migratory effect of exosomes isolated from irradiated cells protein function analysis assigned the deregulated exosomal proteins to cell motility and AKT-signalling. Together, our findings demonstrate that exosomes derived from irradiated HNSCC cells confer a migratory phenotype to recipient cancer cells. This is possibly due to radiation-regulated exosomal proteins that increase AKT-signalling. We conclude that exosomes may act as driver of HNSCC progression during radiotherapy and are therefore attractive targets to improve radiation therapy strategies.

## Introduction

Radiotherapy is a widely used treatment modality for head and neck cancer. However, radiation resistance, local recurrence as well as distant metastasis are commonly encountered treatment complications^1^. There are indications that the radiation treatment itself may increase the motility of glioblastoma, lung and head and neck cancer cells, thus influencing invasion capacity and the migration to local and distant sites^2–4^. In accordance, head and neck cancer patients had a significant higher incidence of distant metastasis if they received preoperative radiotherapy, although the overall survival was not affected^5^. Furthermore, *in vitro* studies found that irradiation increased cellular migration in head and neck cancer cell lines^6,7^. These findings suggest that radiation may promote the acquisition of a more motile phenotype in head and neck cancer cells. However, neither key components nor the underlying mechanisms of this phenomenon are fully understood.

Exosomes are a candidate to stimulate local tumour cell movement and pre-metastatic niche formation^8,9^. Exosomes are nanometer-sized, extracellular vesicles that are released from almost all cell types through the fusion of endosomal multivesicular bodies (MVBs) with the plasma membrane. They contain a variety of biomolecules including RNA, DNA, lipids and several different classes of proteins (e.g. signalling molecules, membrane trafficking proteins, cytoskeleton proteins, adhesion molecules, chaperones, enzymes)^10^. Protein loading is regulated by endosomal sorting complexes required for transport (ESCRT), tetraspanins and lipid-mediated processes, while RNA loading seems to depend on specific sequence motifs and interaction with RNA-binding proteins^11^. Cellular stress, including ionizing radiation, induces changes in the abundance of these exosomal molecules^12–14^.

Released exosomes can interact with recipient cells either by ligand-receptor interaction and induction of intracellular signalling pathways after surface attachment or they can be incorporated by endocytosis or direct fusion resulting in the delivery of their cargo^15,16^. Subsequently, the exosomal cargo is functional within recipient cells and can modify their physiological state^17–20^.

**Figure 1:**
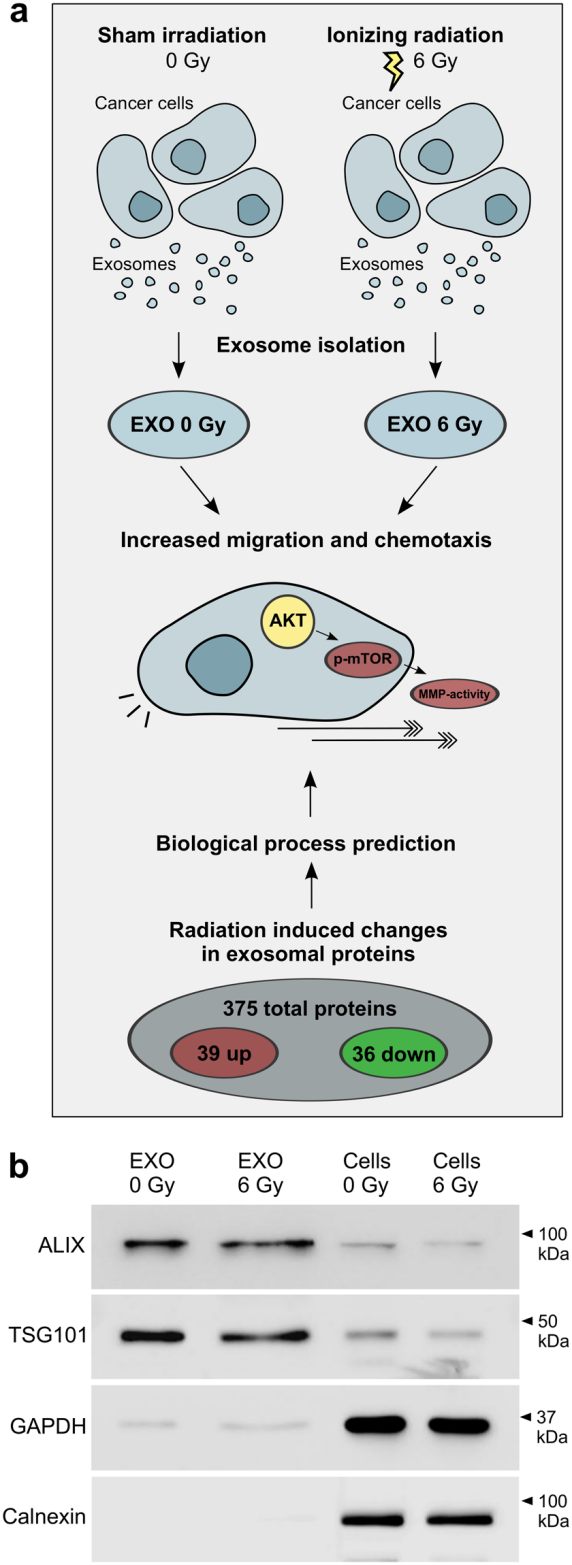
Functional and molecular comparison of exosomes released from 6 Gy irradiated and non-irradiated head and neck cancer cells. Exosomes isolated from irradiated BHY cells induce migration and chemotaxis by activating AKT-signalling and extracellular MMPs. In the same line radiation-induced changes of exosomal proteins predict effects on migration, chemotaxis and AKT-signalling. (**b**) Representative, cropped western blot of exosome markers ALIX and TSG101 as well as cytosolic markers GAPDH and Calnexin for BHY exosomes and cells isolated 24 hours after 0 and 6 Gy irradiation.

In a previous study we have demonstrated that exosomes modulate the radioresistance of head and neck cancer cells, indicated by higher survival and accelerated DNA repair in cells treated with exosomes isolated from irradiated cells^21^. Addressing the clinically relevant observation of radiation effects on local tumour recurrence and metastasis, we investigated if exosomes released from irradiated and non-irradiated cells differentially affect the migratory potential of HNSCC cells and if the radiation-induced changes in the exosomal cargo may trigger these effects (Fig. 1a).

## Results

### Exosomes from irradiated cells promote migration and increase chemotaxis-induced motility

Exosomes were isolated from the conditioned medium of irradiated or non-irradiated BHY squamous head and neck carcinoma cells by differential ultracentrifugation. Exosomes either purified from irradiated (EXO 6 Gy) or non-irradiated (EXO 0 Gy) cells showed the expected enrichment of the exosome marker proteins ALIX and TSG101 over cellular lysates. GAPDH was weakly detected in exosome lysates while it was highly abundant in cellular fractions. Calnexin, a protein not present within exosomes, was absent in exosome lysates, but showed a strong abundance in the cellular lysates (Fig. 1b). Furthermore transmission electron microscopy and nanoparticle tracking analysis confirmed homogeneous exosome preparations with a major population at an average size of 100–130 nm (Supplementary Fig. S1).

**Figure 2:**
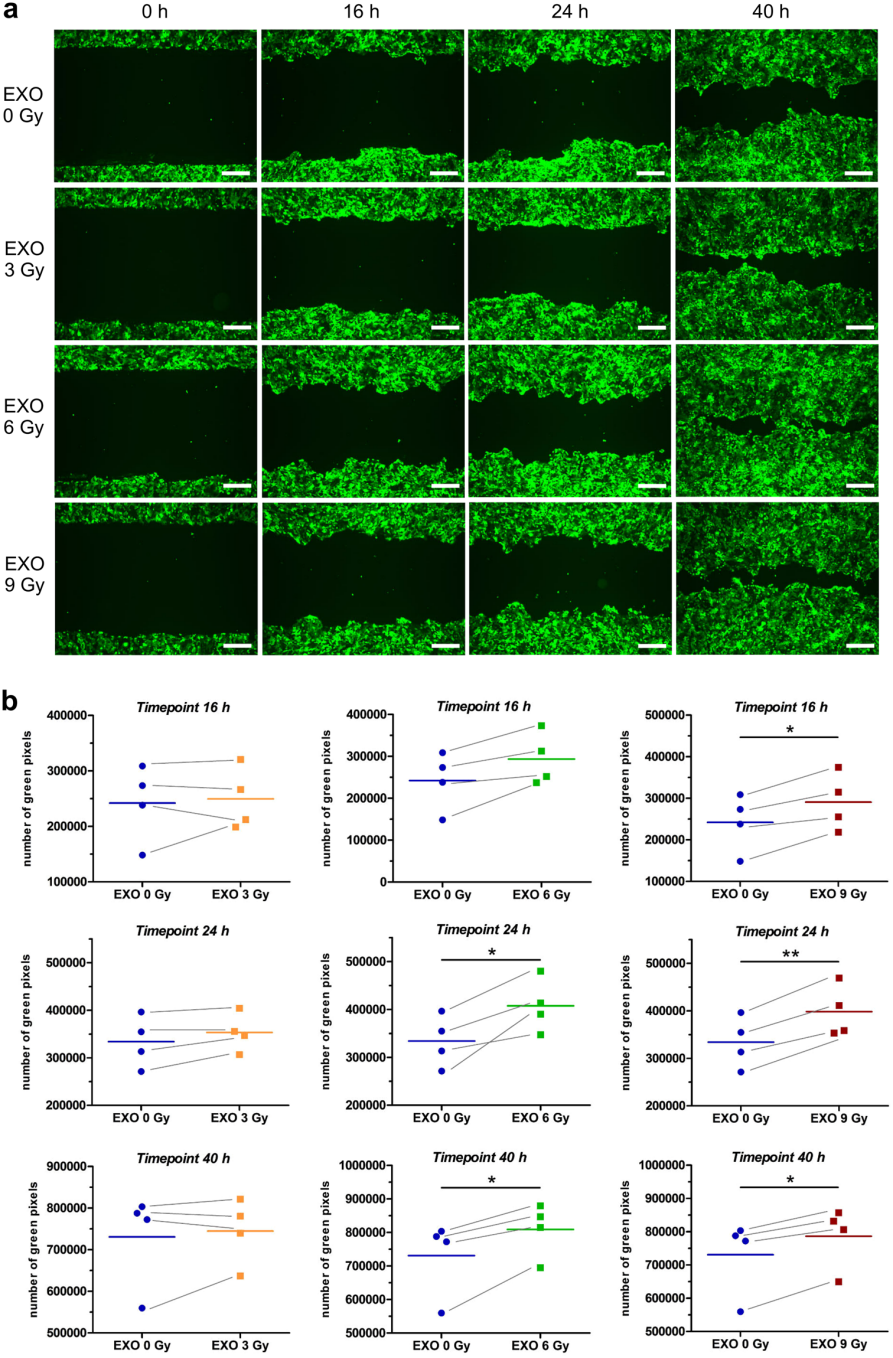
Exosomes from irradiated BHY cells enhance the migratory phenotype. (**a**) Exemplary wound healing of BHY-GFP cells after 16, 24 and 40 hours (scale bar: 500 µm). Cells were either preincubated with exosomes from non-irradiated (EXO 0 Gy), 3 Gy (EXO 3 Gy), 6 Gy (EXO 6 Gy) or 9 Gy (EXO 9 Gy) irradiated BHY cells. (**b**) Quantification of the wound healing capacity with the Image Colour Analyser after 16, 24 and 40 hours [n = 4; two-sided, paired t-test; p-value < 0.05].

To study the influence of exosomes on cell migration we performed a gap-closure assay. BHY cells expressing green fluorescent protein (BHY-GFP) were preincubated with BHY exosomes isolated from either non-irradiated (EXO 0 Gy) or irradiated (EXO 3 Gy, EXO 6 Gy, EXO 9 Gy) cells. Figure 2a depicts a time course of the cellular movement of BHY-GFP cells. Cells preincubated with exosomes isolated from 6 Gy (EXO 6 Gy) and 9 Gy (EXO 9 Gy) irradiated cells closed the gap faster than cells incubated with exosomes from non-irradiated cells, indicating a migration stimulatory effect of exosomes from the irradiated cells. A lower radiation dose of 3 Gy (EXO 3 Gy) did not result in an enhanced migration, indicating that a pro-migratory response of exosomes is dose-dependent for head and neck cancer cells (Fig. 2a and b). To test if the observed exosome-stimulated migration is a ubiquitous phenomenon for head and neck cancer, we analysed the migratory behaviour of FaDu head and neck cancer cells after exosome incubation. Exosomes from irradiated FaDu cells boosted the migration of FaDu-GFP cells compared to exosomes from non-irradiated cells (Supplementary Fig. S2). Exosomal crosstalk between BHY and FaDu was studied by analysing the effect on the migration potential after exosome cross-transfer. Indeed, exosomes isolated from irradiated FaDu cells induced the migration of BHY-GFP cells and exosomes from irradiated BHY cells increased the motility of FaDu-GFP cells (Supplementary Fig. S2). Furthermore, we studied the radiation-induced migration effect of exosomes derived from non-tumour cells. Exosomes isolated from irradiated fibroblasts increased the motility of BHY-GFP cells, but to a lesser extent than exosomes from irradiated head and neck cancer cells. However, exosomes isolated from endothelial cells did not affect the migratory behaviour of BHY-GFP cells (Supplementary Fig. S2).

**Figure 3:**
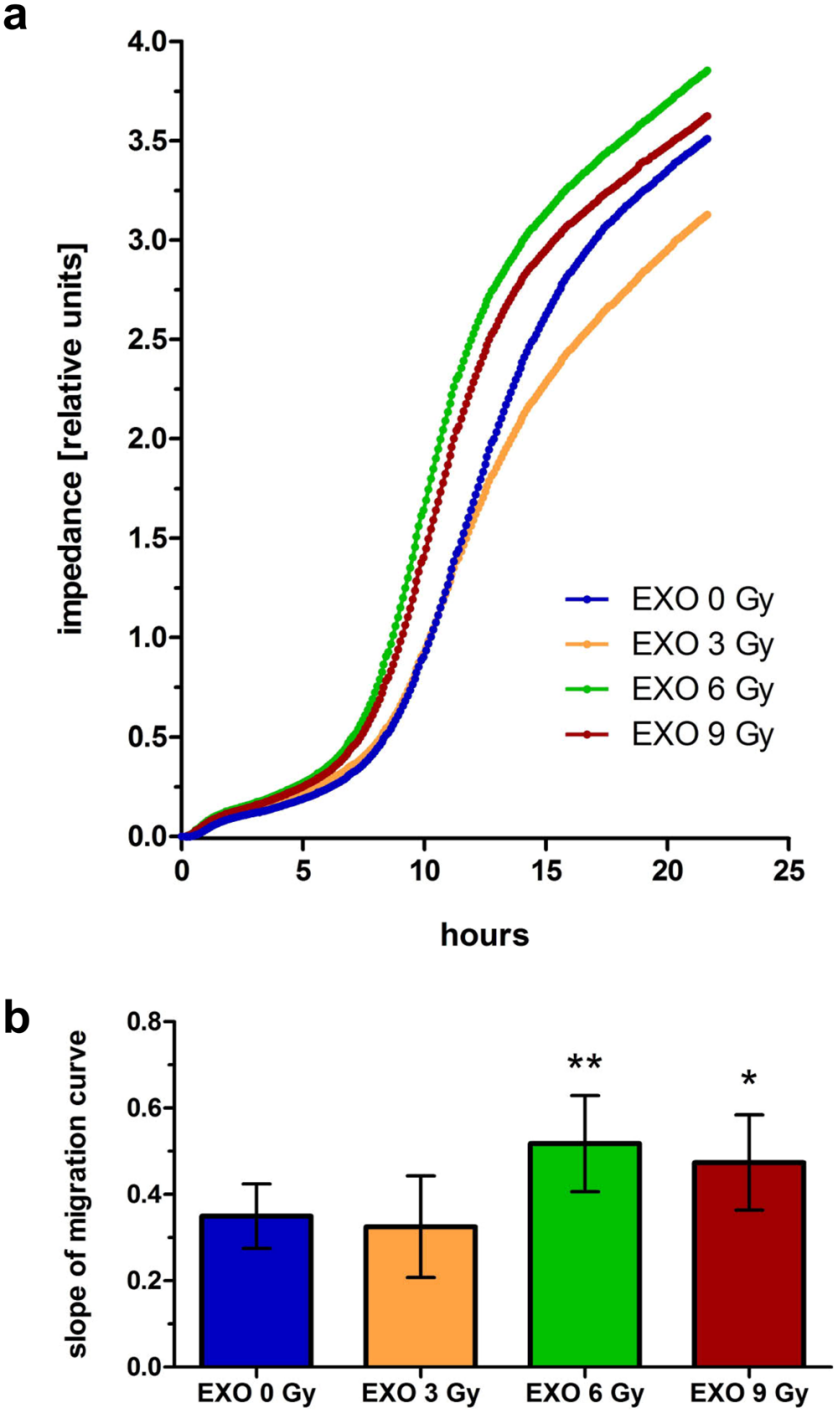
Exosomes from irradiated BHY cells enhance the chemotaxis-induced motility. The xCELLigence system was used to analyse the chemotactic movement of cells after a 24 hours pretreatment with exosomes from non-irradiated (EXO 0 Gy), 3 Gy (EXO 3 Gy), 6 Gy (EXO 6 Gy) or 9 Gy (EXO 9 Gy) irradiated BHY cells. (**a**) Mean impedance as measure of transfilter migration of cells is plotted over time. (**b**) Slope of the migration curves [n = 3; ± SD; two-sided, unpaired t-test; *p-value < 0.05; **p-value < 0.01].

Additionally, we examined if exosomes are influencing motility by altering chemotaxis. The impedance, as a measure of transfilter migration, was more rapidly increased for BHY cells incubated with exosomes isolated from 6 Gy and 9 Gy irradiated BHY cells in comparison to cells treated with exosomes from non-irradiated cells (Fig. 3a). The slope of the migration curve confirmed that these exosomes augment the chemotactic phenotype (Fig. 3b). In contrast, exosomes from 3 Gy irradiated cells did not affect the chemotactic motility (Fig. 3a and b).

### Exosomes from irradiated head and neck cancer cells trigger the AKT-pathway

**Figure 4:**
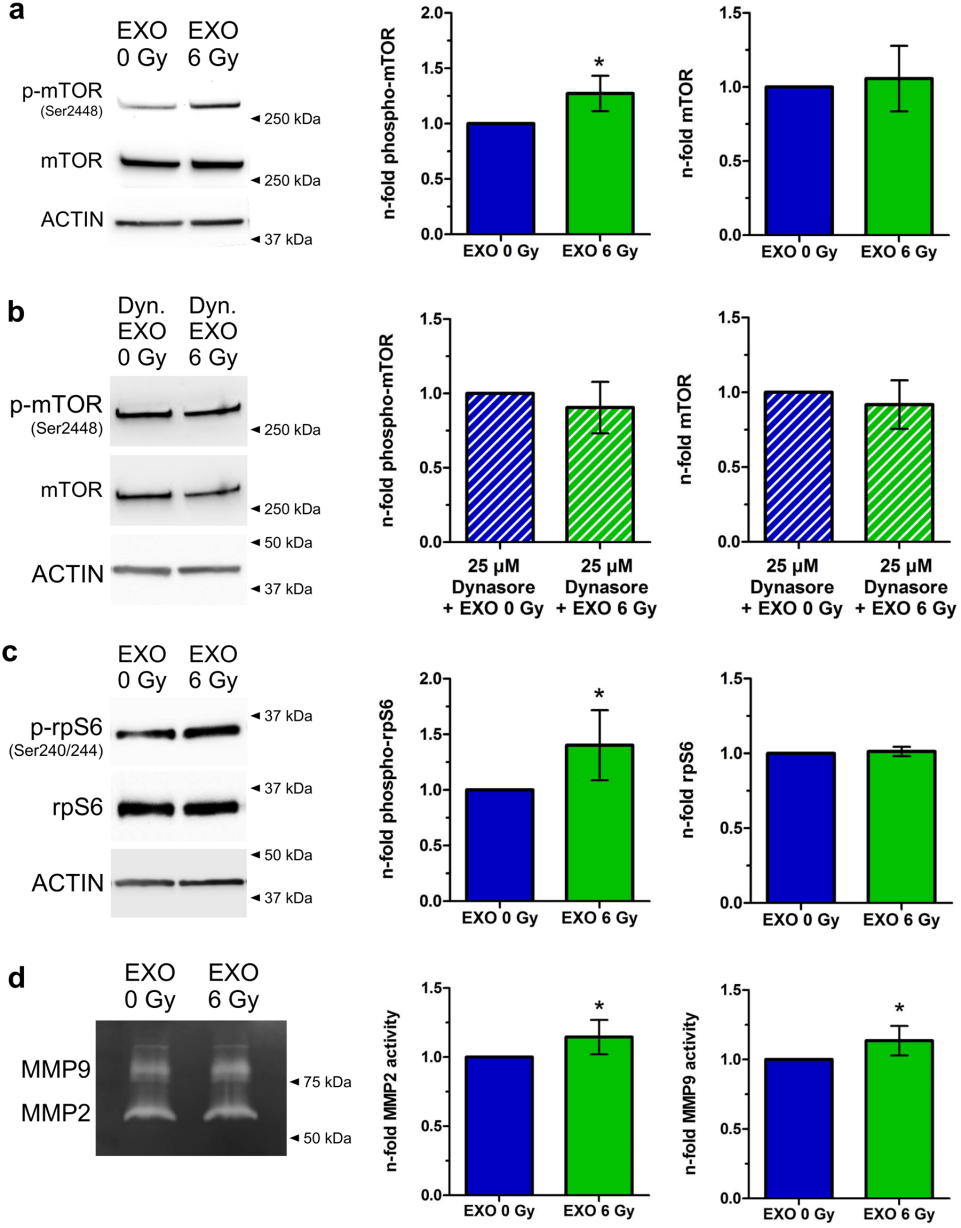
Exosomes from irradiated cells activate the AKT-pathway. (**a**) Western blot of phospho-mTOR (Ser2448) and mTOR of cells which were incubated for 24 hours with exosomes isolated either from irradiated cells (EXO 6 Gy) or from non-irradiated cells (EXO 0 Gy). Normalization was performed to ACTIN and to cells treated with exosomes from non-irradiated cells (EXO 0 Gy). Cropped blots are displayed [n = 4; ± SD; two-sided, one-sample t-test; p-value < 0.05]. (**b**) Western blot of phospho-mTOR (Ser2448) and mTOR of cells which were pretreated for 1 hour with 25 µM Dynasore and incubated for 24 hours with exosomes isolated either from irradiated cells (EXO 6 Gy) or from non-irradiated cells (EXO 0 Gy). Normalization was performed to ACTIN and to cells treated with exosomes from non-irradiated cells (EXO 0 Gy). Cropped blots are displayed [n = 3; ±SD; two-sided, one-sample t-test]. (**c**) Western blot of phospho-S6 Ribosomal Protein (Ser240/244) and S6 Ribosomal Protein of cells which were incubated for 24 hours with exosomes isolated either from irradiated cells (EXO 6 Gy) or from non-irradiated cells (EXO 0 Gy). Normalization was performed to ACTIN and to cells treated with exosomes from non-irradiated cells (EXO 0 Gy). Cropped blots are displayed [n = 7; ±SD; two-sided, one-sample t-test; p-value < 0.05]. (**d**) MMP2 and MMP9 activity in the supernatants 24 hours after transfer of exosomes isolated from irradiated (EXO 6 Gy) and from non-irradiated cells (EXO 0 Gy) on BHY cells. Normalization was performed to cells treated with EXO 0 Gy. Cropped gels are displayed [n = 6; ±SD; two-sided, one-sample t-test; p-value < 0.05].

One key regulator of migration processes in head and neck cancer is AKT-signalling^22,23^. To examine a potential effect of exosomes on AKT-pathway regulation the downstream target mTOR was analysed after 3 and 24 hours of exosome incubation. mTOR is predominantly phosphorylated at Ser2448 in response to stimuli which activate AKT^24^ and is a mediator of pro-migratory signals in head and neck cancer^25–27^. The phosphorylation on Ser2448 of mTOR was increased at both time points after transfer of exosomes isolated from 6 Gy irradiated cells, compared to exosomes from non-irradiated cells (Fig. 4a, Supplementary Fig S3). This effect can be abrogated if endocytosis of exosomes is inhibited by Dynasore (Fig. 4b).

Furthermore, the phosphorylation level on Ser240/244 of S6 Ribosomal Protein (rpS6), a downstream target of the mTOR-signalling^28^, was increased 24 hours after transfer of exosomes isolated from irradiated cells (Fig. 4c).

The increased motile phenotype of head and neck cancer cells receiving exosomes from irradiated cells was accompanied by increased matrix metalloproteinase (MMP) activity. MMP2 and MMP9 are both downstream targets of the AKT signalling and drive cellular motility^29–31^. Cells treated with exosomes from 6 Gy irradiated cells, released significantly more MMP2 and MMP9 in the supernatant, compared to cells supplemented with exosomes from non-irradiated cells (Fig. 4d).

The increase in mTOR-, rpS6-phosphorylation and MMP activity suggest that exosomes from irradiated cells are able to activate the AKT-signalling pathway in recipient cells.

### AKT-pathway is required for exosome-mediated migration after ionizing radiation

**Figure 5:**
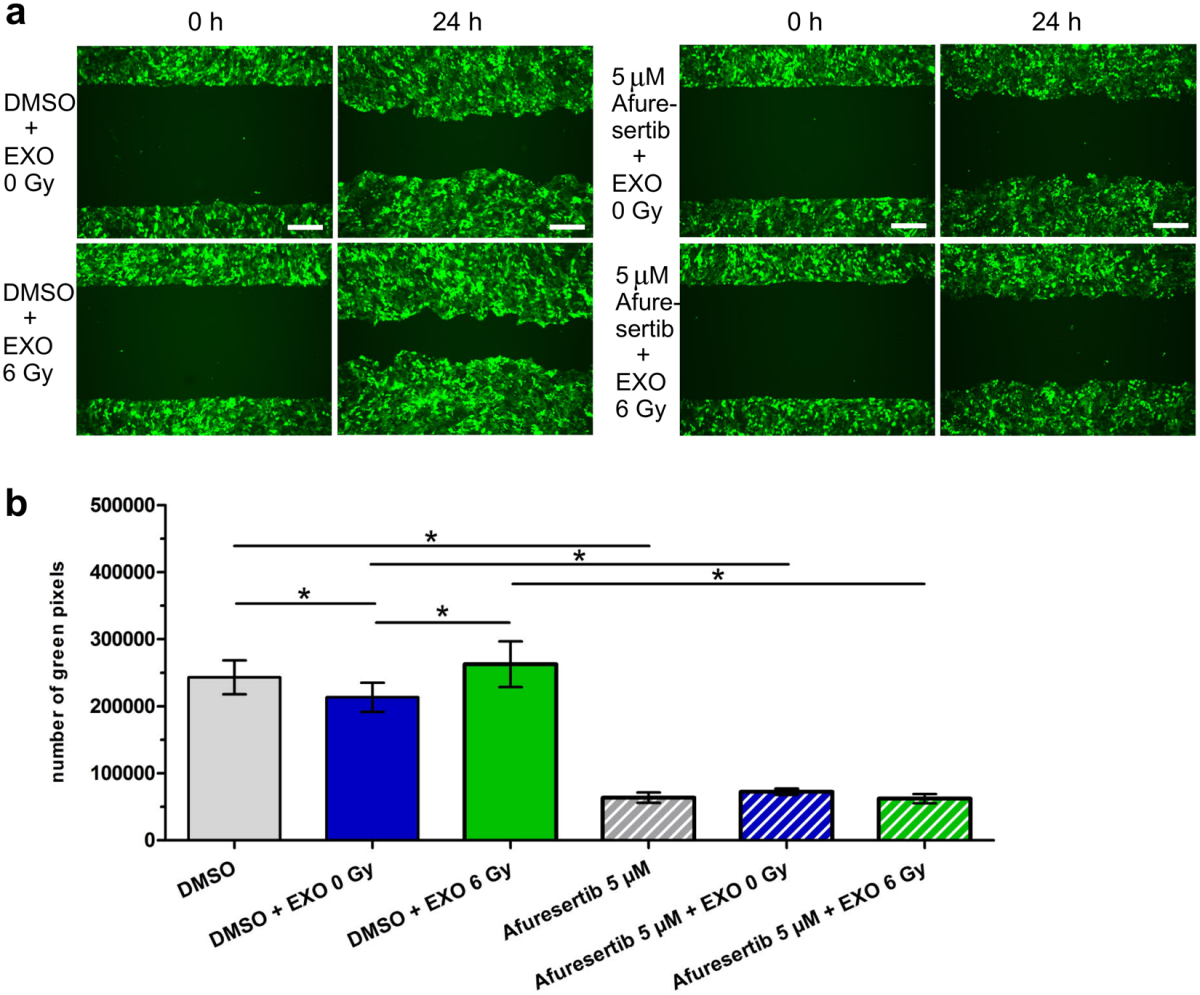
Exosomes from irradiated cells activate the AKT-pathway to induce migration. (**a**) Exemplary wound healing of BHY-GFP cells after treatment with 5 µM of the AKT-inhibitor Afuresertib or DMSO, in combination with exosomes isolated from irradiated (EXO 6 Gy) and from non-irradiated cells (EXO 0 Gy). The pictures were taken 24 hours after migration start (scale bar: 500 µm). (**b**) Quantification of wound healing capacity with the Image Colour Analyser 24 hours [n = 3; ±SD; two-sided, paired t-test; p-value < 0.05].

The AKT-inhibitor Afuresertib was used to block AKT activity. Indeed treatment with 5 µM Afuresertib caused reduced levels of phosphorylated mTOR, confirming that Ser2448 phosphorylation of mTOR is triggered by AKT in BHY cells (Supplementary Fig. S3). Moreover AKT-inhibition reduced the migration of BHY-GFP cells in comparison to the control DMSO-treated cells (Supplementary Fig. S3). Combination of Afuresertib with exosome incubation was able to prevent the pro-migration effect of exosomes isolated from 6 Gy irradiated cells (Fig. 5a and b). Inhibition of AKT with Afuresertib reduced the activity of MMP2 and MMP9, indicating that the MMP activity is AKT-dependent in BHY cells (Supplementary Fig. S3).

### Exosomes from donor head and neck cancer cells transfer proteins to recipient cells

**Figure 6:**
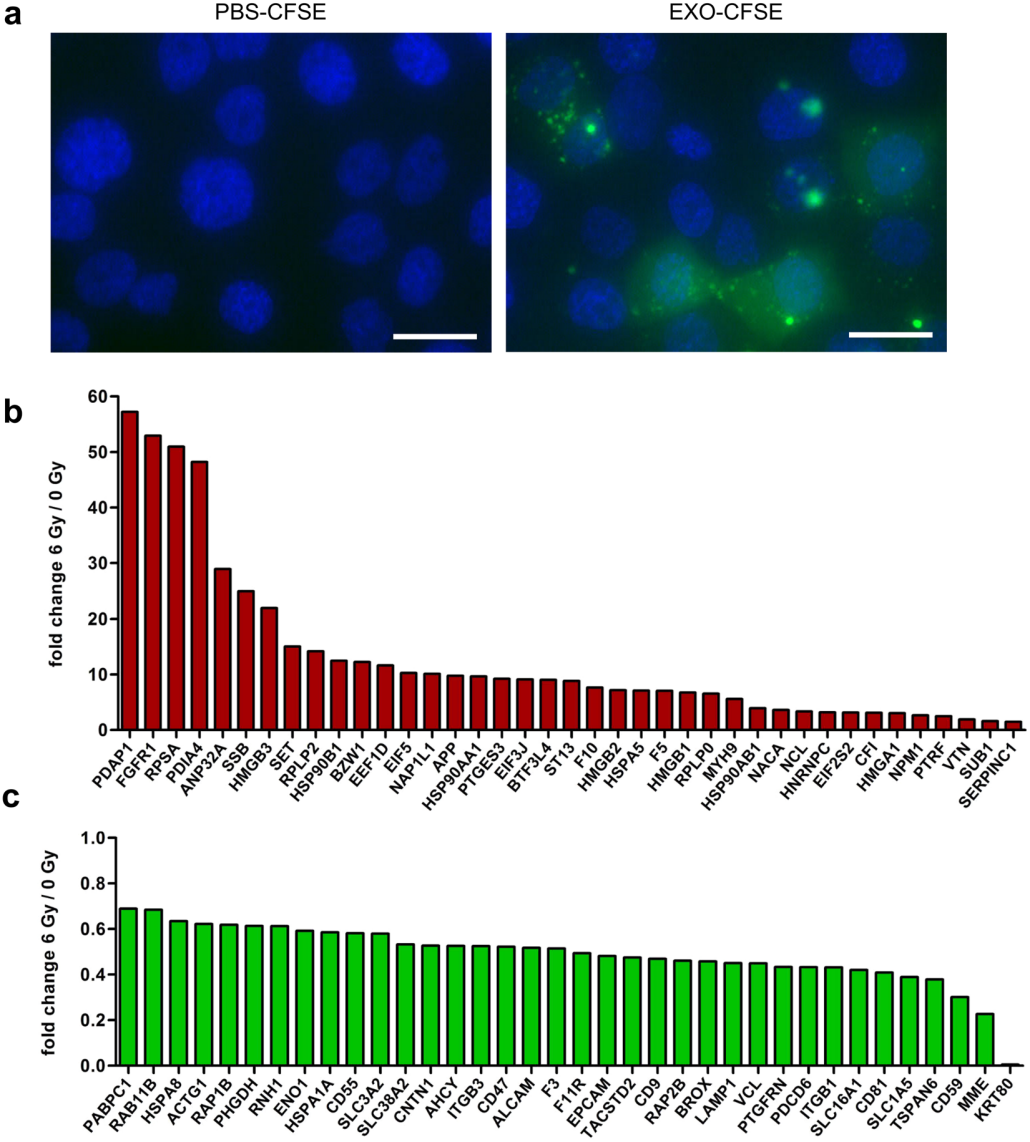
Exosomes from head and neck cancer cells transfer proteins to recipient cells and have a modified protein composition after ionizing radiation. (**a**) Exosomal proteins (EXO-CFSE) of BHY cells and PBS (PBS-CFSE) as negative control were stained with CFSE and subsequently transferred onto recipient BHY cells. The protein uptake was monitored after 24 hours of exposure (scale bar: 25 µm). Protein analysis of exosomes isolated 24 hours after 6 Gy irradiation of the head and neck cancer cell line BHY revealed (**b**) 39 upregulated and (**c**) 36 downregulated proteins [n = 3; FDR-adjusted p-value (q-value), ≥2 unique peptides, fold-change between ≤0.7 and ≥1.3; q-value < 0.05.

To further understand the role of exosomes in modifying migration capacity of recipient cells, we studied their ability to transfer exosomal proteins. BHY-derived exosomes were labelled with carboxyfluorescein succinimidyl diacetate ester (CFSE) and added to recipient BHY cells. Uptake and cytoplasmic distribution of the labelled proteins was visible 24 hours after exosome transfer to recipient cells, confirming that exosomes serve as an efficient tool for protein exchange between BHY cells (Fig. 6a, Supplementary Video S1). The control, PBS plus CFSE, did not display any fluorescence. The preincubation with the dynamin inhibitor Dynasore blocked the protein uptake, which suggests endocytosis as major exosome uptake mechanism in BHY cells (Supplementary Fig S4). We also studied exosome communication between different cell lines. Indeed FaDu cells took up BHY exosomal proteins and BHY cells absorbed exosomal proteins derived from FaDu cells. Furthermore, exosomal proteins from non-tumour fibroblasts and endothelial cells were transferred to both head and neck cancer cell lines (Supplementary Fig. S4). Exosomes are therefore potent vehicles to transfer proteins between same and different cell types.

### Exosomal proteome of BHY cells

In order to examine whether exosome cargo contains proteins involved in induction of cell migration, we analysed exosomes that were isolated from BHY cells 24 hours after irradiation with 0 or 6 Gy with label-free quantitative proteomics. A total of 375 proteins were detected in the isolated exosomes. All proteins identified in BHY exosomes are listed in Supplementary Table S1. The detected proteins are grouped by STRING software *in silico* analysis into the compartments ‘extracellular vesicle’ and ‘extracellular exosome’ with a false discovery rate (FDR) of 1.1e^−156^–3.5e^−156^ (Supplementary Table S2). A comparison between all identified exosomal proteins of BHY cells and the top 50 (most often detected) exosomal proteins listed in the ExoCarta global exosomal protein database showed an overlap of 86% (Supplementary Table S3). These findings support the conclusion about a conserved subset of exosomal proteins across cell types.

### The composition of the exosomal protein cargo is modified following exposure to ionizing radiation

The comparison of exosomes isolated from non-irradiated donor cells (EXO 0 Gy) and exosomes isolated from irradiated donor cells (EXO 6 Gy) revealed that exposure to ionizing radiation modifies the protein content of exosomes secreted by the head and neck cancer cell line BHY. We found 39 proteins up- and 36 proteins downregulated in exosomes isolated from irradiated donor cells compared to exosomes isolated from non-irradiated cells (q-value < 0.05). All deregulated proteins are depicted in Fig. 6b and c and are listed in Supplementary Table S4. Remarkably, several proteins were highly upregulated with a maximal enrichment up to 57-fold.

### *In silico* analysis of radiation-regulated exosomal proteins

STRING analysis on protein function, as based on the number of network edges (196 compared to 77 for a random set of proteins; PPI (protein-protein-interaction) enrichment p-value < 1 × 10^−15^), revealed a high degree of protein interaction amongst the deregulated proteins (Supplementary Fig. S5). This suggests cooperative functions of the deregulated proteins. Indeed, the radiation-regulated exosomal proteins have a predicted influence on 142 biological processes (Supplementary Table S5). A considerable number of identified processes relate to cellular motility. Wound healing (FDR = 3.81e^−9^), locomotion (FDR = 0.0002), biological adhesion (FDR = 0.0004), regulation of cellular component movement (FDR = 0.0005), chemotaxis (FDR = 0.0005) and regulation of cell motility (FDR = 0.0006) were highly predicted to be influenced by the deregulated exosomal proteins released by irradiated cells. In addition Cytoscape pathway enrichment analysis of the deregulated proteins predicted an influence on PI3K-AKT-signalling (FDR = 0.0071) (Supplementary Table S6). Taken together, these results suggest that the radiation-deregulated exosomal proteins may play a role in inducing cellular motility via AKT activation.

## Discussion

Radiation therapy may increase the invasive and metastatic properties of head and neck tumours^5–7^. In this study, we show that exosomes isolated from irradiated squamous head and neck cancer cells promote AKT-dependent migration and chemotaxis-induced motility in recipient cancer cells. Analysis of the exosomal cargo suggests that radiation-induced changes in the exosomal proteins increase migration via the AKT-pathway. As a consequence exosome-mediated cell-to-cell communication during radiotherapy may promote cancer cell motility.

To improve metastasis-free survival, it is essential to understand the underlying mechanism of radiation-induced cell migration. Our data demonstrate that exosomes from irradiated donor cells boost the motility in head and neck cancer cells. Interestingly this effect depends on the irradiation dose applied to the exosome donor cells and suggests dose-dependent alterations in the exosome-mediated cell-to-cell communication. Importantly, migration effects cannot be assigned to differences in proliferation capacity, since we showed equal effects on proliferation of recipient cells which were treated with exosomes isolated from irradiated compared to non-irradiated donor cells^21^. In accordance Arscott *et al*. showed augmented migration of glioblastoma cells after pretreatment or chemotactic stimulation with exosomes isolated from 4 Gy irradiated cells^32^.

Additional evidence for the motility promoting effect of exosomes from irradiated cells comes from our observations that exosomes isolated from irradiated donor cells trigger the AKT-pathway in the recipient cells (evidenced by increased p-mTOR and p-rpS6). The AKT-pathway is the most frequently mutated oncogenic pathway in head and neck cancer, a key regulator of radiation resistance and a major driver of cellular movement and migration processes^22,33–35^. The impact of AKT-signalling on the migration process was confirmed by AKT-inhibition with Afuresertib. Inhibitor treated cells exhibit a reduced, but still existing migratory potential. The observation that exosomes from irradiated donor cells were incapable to compensate the effect of Afuresertib supports the key-role of the AKT-pathway as a regulator of exosome-stimulated migration after ionizing radiation. In line with this, a study from Pickhard *et al*. showed that inhibition of PI3K and mTOR activity with LY294002, respectively rapamycin, blocks the radiation-induced migration of BHY head and neck cancer cells^7^. Moreover, preclinical models and clinical trials already demonstrated that AKT- and mTOR-inhibitors are promising antitumour agents, which might increase the efficacy of radiotherapy and therefore patient survival^22,36^.

AKT induces migration processes through the regulation of MMP activity, which is critical for the degradation of the extracellular matrix^37,38^. Dysregulation of MMP2 and MMP9 is frequently present in head and neck cancers and is associated with lymph node metastasis and poor prognosis^39,40^. Moreover, Park *et al*. identified ionizing radiation as the trigger for increased AKT-pathway induction combined with enhanced MMP2 activity in glioma cells^29^. We have found more active MMP2 and MMP9 to be released after incubation with exosomes from irradiated cells. This supports our suggestion that enhanced AKT-signalling promotes the increased migration through degradation of the extracellular matrix by fine-tuning MMP activity. In a clinical context MMP2 and MMP9 overexpression may be helpful markers in diagnosing head and neck cancer metastasis^41^.

Previous studies showed that external stimuli and stress conditions, including ionizing radiation, are reflected by changes in the exosome composition^14,32,42^. Our proteomic analysis also revealed that radiation induces changes in the exosomal protein content. According to bioinformatics analysis these protein changes have the potential to influence migration processes as well as AKT-signalling in recipient cells. Based on our CFSE labelling results and on previous findings that demonstrated the transfer of proteins by exosomes and their influence on cell fate in the recipient cells^17,18,20^, we suggest that exosomes from irradiated cells might transfer proteins to recipient cells that increase cellular motility by AKT activation and MMP release. The abrogation of increased p-mTOR levels after incubation with EXO 6 Gy and simultaneous blockage of exosome internalization by dynamin-inhibition suggests that rather the transfer of cargo than exosome surface interactions induce the observed effects.

Candidate proteins which were upregulated in exosomes after irradiation, that activate AKT, stabilize MMP2^43^, enhance exosome-mediated motility^44^ as well as metastasis^45,46^ and invasion^47^ are FGFR1, HSP90AA1, HSP90AB1, HSP90B1 and VTN. The second most upregulated protein FGFR1 (53-fold) is overexpressed in 75% of HPV-negative patients with HNSCC, correlates with poor overall and disease-free survival^48^, increases the metastatic potential^45^ and induces radiation resistance^49^. Nonetheless, a cooperative function of several exosomal proteins is highly conceivable.

In summary, exosomes derived from irradiated head and neck cancer cells are able to confer a migratory phenotype to recipient cancer cells via increased AKT-signalling. Our proteomic data suggest a subset of radiation-regulated exosomal proteins as candidates to induce the pro-migratory effects, however we cannot exclude effects of other exosomal components. In a clinical view exosome-mediated cell-to-cell communication may act as potential driver of metastatic head and neck cancer progression during tumour radiation treatment and therefore represents an attractive target to improve radiation therapy strategies.

## Materials and Methods

### Cell lines and irradiation

The human cell lines BHY (DSMZ no.: ACC 404) and FaDu (ATCC®HTB43^TM^) are squamous cell carcinoma of the head and neck region. BHY cells were cultivated in DMEM (Dulbecco’s modified Eagle’s medium, Gibco) with high Glucose, 2 mM L-Glutamine and sodium pyruvate at 10% CO_2_, whereas FaDu cells were cultivated in DMEM (GE Healthcare) with low glucose, 2 mM L-Glutamine and 25 mM HEPES at 5% CO_2_. For both cell lines medium was supplemented with 10% FCS (foetal calf serum, Bio&SELL). The human skin fibroblast cells 1BR3 (ECACC 90011801) were maintained in DMEM with low glucose and 15% FCS at 5% CO_2_. The human coronary artery endothelial cells HCAEC (ATCC® PCS-100–020™) were cultivated in MesoEndo Cell Growth Medium Kit (Cell Applications) at 5% CO_2_. All cells were incubated in a humidified atmosphere at 37 °C.

BHY-GFP and FaDu-GFP cells (expressing green fluorescence protein) were established by lentiviral transduction using pGreenPuro transfer vector (SBI, CA, USA) and previously described lentiviral protocols^50,51^. For stable and constitutive GFP expression cells were cultivated in DMEM medium containing 0.3 µg/ml or 0.1 µg/ml puromycine for BHY-GFP or FaDu-GFP cells, respectively.

For AKT-inhibition BHY or BHY-GFP cells were treated for 3 or 24 hours with 5 µM of Afuresertib (GSK2110183; Cell Signaling Technology). The Dynamin inhibitor Dynasore (25 µM, CAS 304448-55-3, Sigma) was added to BHY cells 1 hour before exosome treatment. Control cells were sham-treated with DMSO (Sigma-Aldrich).

Cell line identification was confirmed by Eurofins Genomics (sequencing of nine different loci: D5S818, D13S317, D7S820, D16S539, VWA, TH01, AM, TPOX, CSF1PO). Mycoplasma negative status was confirmed with MycoAlert.

A ^137^caesium source (HWM-D2000, Wälischmiller Engineering) was used to irradiate the cells with γ-rays at a dose rate of 0.45 Gy per min.

### Isolation of exosomes

Exosomes were isolated from culture supernatants by a serial centrifugation procedure as previously described^21^. Briefly, cells were seeded in 10 cm dishes and fresh medium with exosome-depleted FCS (edFCS) was added prior to irradiation. After 24 hours of cultivation, the medium was collected, centrifuged at 10,000 g for 30 minutes at 4 °C and passed through a filter with a pore size of 0.22 µm. The filtrate was centrifuged with 100,000 g for 75 minutes at 4 °C. The supernatant was discarded and the exosome pellet was resuspended in PBS. Another round of ultracentrifugation (100,000 g, 75 minutes, 4 °C) was applied and the final exosome pellet resuspended in fresh PBS. To determine the biological activity of exosomes we incubated recipient cells with exosomes in medium supplemented with edFCS and exosome preparations isolated from irradiated and non-irradiated donor cells. Applied exosome amounts correspond to a three-fold concentration of exosomes compared to native conditions. Exosomes were stored at −20 °C. Cells were harvested using a cell scraper and stored at −20 °C.

For the preparation of edFCS, bovine exosomes were removed from foetal calf serum by centrifugation at 100,000 g and 4 °C for 14 hours.

### Electron microscopy

BHY and FaDu exosomes (isolated from 3 ml conditioned medium) were absorbed onto glow discharged carbon coated grids (G2400C from Plano) for 2 minutes. The solution was blotted of and negatively stained with 4% ammonium molybdate (Sigma-Aldrich) solution for 30 seconds. Micrographs were recorded with a Jeol JEM 100CX electron microscope at 100 kV onto Kodak SO163 film. Negatives were digitized with a Hasselblad Flextight × 5 scanner at 3000 dpi, resulting in a pixel size of 0.25 nm/px. For visualization images were binned to 1 nm/px.

### Exosome size

Exosome size distribution was analysed by using the NanoSight LM10 (Malvern) microscope. Exosome preparations (isolated from 2.5 ml conditioned medium) were diluted 1:600 with H_2_O to achieve 15 to 50 particles per frame for tracking. Each sample was analysed three times for 30 seconds.

### Migration assay

Gap-closure (wound healing) was performed with GFP labelled cells. Silicon grids (Ibidi) with 12 rectangular wells and a wall size of 2 mm were placed air bubble-free in 10 cm cell culture dishes. 42,000 BHY-GFP or 60,000 FaDu-GFP cells were then seeded per well. After cell attachment the medium was discarded and replaced by exosome-depleted medium. Subsequently cells were pretreated with exosomes, 5 µM of the AKT-inhibitor Afuresertib (GSK2110183; Cell Signaling Technology) or DMSO (Sigma-Aldrich). After 24 hours the medium was discarded and the silicon grids were removed carefully to generate a defined gap (2 mm) in the monolayer. 8 ml of exosome-depleted medium, medium containing 5 µM Afuresertib or DMSO were added. Starting pictures (0 hour) were taken immediately after grid removal and repeated after 16, 24, 40 and 48 hours to monitor migration. For quantification Adobe Photoshop CS5 (Adobe Systems) was used to identify green fluorescent cells from the starting picture (0 hour) and to subtract this area in pictures from later time points. Finally the program Image Colour Analyser (developed by Marcus Vetter; source code available upon request) was used to quantify the migratory potential. This tool analyses the green colour value in an intensity range from 0 to 255 (RGB-range) for each pixel of the picture and allows the calculation of the total pixel number that exceed a given green value.

### Chemotaxis-induced motility

The xCELLigence® Real-Time Cell Analyser (RTCA) DP System (Roche) was used to measure gradient-driven cell movement. BHY cells were pretreated for 24 hours with exosomes in medium containing edFCS. Then cells were re-plated into CIM-plates (Roche Diagnostics) with 8 μm pores. In total 60,000 cells in 1% edFCS-containing medium were seeded into the upper chamber, while the lower chamber contained 10% edFCS as chemoattractant. Chemotaxis-induced migration was tracked in real-time over a time span of 24 hours in the RTCA DP instrument at 37 °C with 10% CO_2_. The increase in impedance measured on electrodes on the lower surface of the filter membrane reflects cell migration^4^.

### Zymography

To measure gelatinase activity, cell culture supernatants were collected 24 hours after exosome or Afuresertib (GSK2110183; Cell Signaling Technology) treatment and concentrated (5-fold) with centrifugal filter units (Amicon Ultra; 0.5; 100k). The BCA-assay (Pierce^TM^ BCA Protein Assay Kit, Thermo Fisher Scientific) was applied according to the manufacturer’s instructions to determine the protein concentration. Equal amounts of protein were treated with 5x non-denaturing sample buffer (4% SDS, 20% glycerol, 0.01% bromophenol blue, 125 mM Tris-HCl) and separated in a 10% polyacrylamide SDS gel containing 1 mg/ml gelatine (Sigma). After electrophoresis, the gel was washed twice with washing buffer (2.5% Triton X-100, 50 mM Tris-HCl, 5 mM CaCl_2_, 1 µM ZnCl_2_) for 30 minutes, rinsed once in incubation buffer (1% Triton X-100, 50 mM Tris-HCl, 5 mM CaCl_2_, 1 µM ZnCl_2_) and stored for 24 hours at 37 °C in the incubation buffer. A 5% Coomassie solution was added for 60 min to stain the gel. Destaining solution containing 40% methanol and 10% acetic acid was applied until gelatine digestion was visible as clear bands against the background. The detection camera FluorChem HD2 (Alpha Innotec) and the Alpha View Software (ProteinSimple) were used to image the gelatine digestion.

### Quantification of exosomal and cellular proteins

Exosomes and cells were disrupted in lysis buffer II (25 mM Tris pH 7.5, 120 mM NaCl, 1% Triton X-100, 1% PSMF, 1 mM NOV, 1 mM Leupeptin) on ice. Exosomes were lysed for 4 hours, while cells were incubated with the lysis buffer II for 1 hour. The protein concentration was determined by applying the BCA-assay (Pierce^TM^ BCA Protein Assay Kit, Thermo Fisher Scientific) according to the manufacturer’s instructions.

For immunoblotting 10 µg cellular protein and 10 µl exosome lysate (isolated from 3.5 × 10^6^ cells) were used to run a standard western blot protocol. Antibodies directed against ALIX (2171, Cell Signaling), TSG101 (GTX70255, GeneTex), GAPDH (sc-47724, SantaCruz), Calnexin (sc11397, SantaCruz), p-mTOR Ser2448 (5536, Cell Signaling Technology), mTOR (2983, Cell Signaling), p-AKT Ser473 (9271, Cell Signaling Technology), p-S6 Ribosomal Protein Ser240/244 (2215, Cell Signaling), S6 Ribosomal Protein (2212, Cell Signaling) and ACTIN (SAB1305567, SIGMA-Aldrich Chemie) were applied. Secondary horseradish peroxidase-conjugated antibodies (1:40.000; anti-rabbit: sc2004 and anti-mouse: sc2005) and the chemoluminescence Amersham ECL reaction kit (GE Healthcare) were used for detection.

### Trafficking of exosomes monitored with fluorescent labelled proteins

To monitor the exosome-mediated trafficking of proteins the Exo-Glow^TM^ kit (System Biosciences), based on carboxyfluorescein succinimidyl diacetate ester (CFSE) chemistry, was applied with slight variation to the manufacturer’s protocol. Exosomes were incubated with 1x Exo-Green for 10 minutes at 37 °C. To remove residual dye the samples were loaded on exosome-spin columns (Invitrogen) and processed according to the manufacturer’s protocol. Exosomes with green fluorescent labelled proteins were transferred onto BHY cells. Nuclei staining was performed 24 hours later by adding NucBlue^TM^ Live Cell Stain (Life Technologies). The uptake of the exosome-mediated proteins was monitored by fluorescence microscopy.

### Quantitative proteomic analysis

Exosomal proteins were isolated by adding 20 µl of lysis buffer II (25 mM Tris pH 7.5, 120 mM NaCl, 1% Triton X-100, 1% PSMF, 1 mM NOV, 1 mM Leupeptin) to 40 µl of exosome suspension isolated from 1.5 × 10^7^ cells. The samples were incubated for 4 hours on ice with repeated vortexing and the protein concentration was determined by the BCA assay (Pierce^TM^ BCA Protein Assay Kit, Thermo Fisher Scientific) following the manufacturer’s instructions.

Sample preparation, LC-MS/MS measurement, label-free quantitative analysis and database searches were performed as previously described^13^. Briefly, 5 µg of protein were digested using a modified filter-aided sample preparation (FASP), followed by the LC-MS/MS analysis performed on a LTQ OrbitrapXL (Thermo Fisher Scientific) coupled to an Ultimate3000 nano high-performance liquid chromatography system (Dionex). Alignment of peptides was set to at least 89.5% and single charged features as well as features with charges higher than +7 were eliminated. The Mascot search engine (Matrix Science, version 2.5.0) with the Ensembl Human database (version 83, 31236148 residues, 83462 sequences) was used for identification.

To identify significantly changed proteins a FDR-adjusted p-value (q-value) of three independent biological replicates was calculated. Here peptides with ≥2 unique peptides, a fold-change between ≤0.7 and ≥1.3 plus a q-value of <0.05 were considered as statistically significant deregulated.

*In silico* analysis was performed with several bioinformatics tools. The top exosomal protein candidates of ExoCarta, the web-based database of exosomal proteins, RNA and lipids, was used to compare the detected exosomal proteins from BHY cells with proteins recorded within exosomes ((http://exocarta.org/exosome_markers_new) accessed 09.03.2017)^52^. Protein subcellular localizations and functions were determined using STRING: *functional protein association networks* (http://STRING-db.org/)^53^. A pathway enrichment analysis (FDR < 0.05) of the deregulated exosomal proteins was performed using the Reactome 5.1.0 application^54^ in the Cytoscape 3.2.1 software^55^.

### Statistical analysis

Data show the mean of independent biological experiments with the standard deviation (±SD). The two-sided paired, unpaired or the one-sample t-test were used for statistical analysis and a p-value < 0.05 was deemed statistically significant, while a p-value < 0.01 was considered highly significant.

### Data availability

The datasets generated during the current study are available from the corresponding author on reasonable request. The MSF files of the obtained MS/MS spectra can be found under STUDY1095 in https://www.storedb.org.

## Electronic supplementary material

Supp Video 1

Supplementary Information

Supp Tables

## References

[1] Neville BW, Day TA (2002). Oral cancer and precancerous lesions. CA Cancer J Clin.

[2] Vilalta M, Rafat M, Graves EE (2016). Effects of radiation on metastasis and tumor cell migration. Cellular and molecular life sciences: CMLS.

[3] Moncharmont C (2014). Radiation-enhanced cell migration/invasion process: a review. Crit Rev Oncol Hematol.

[4] Edalat L (2016). BK K+ channel blockade inhibits radiation-induced migration/brain infiltration of glioblastoma cells. Oncotarget.

[5] Strong MS (1978). A randomized trial of preoperative radiotherapy in cancer of the oropharynx and hypopharynx. Am J Surg.

[6] Beck C (2012). The kallikrein-kinin-system in head and neck squamous cell carcinoma (HNSCC) and its role in tumour survival, invasion, migration and response to radiotherapy. Oral Oncol.

[7] Pickhard AC (2011). Inhibition of radiation induced migration of human head and neck squamous cell carcinoma cells by blocking of EGF receptor pathways. BMC cancer.

[8] Sakha S, Muramatsu T, Ueda K, Inazawa J (2016). Exosomal microRNA miR-1246 induces cell motility and invasion through the regulation of DENND2D in oral squamous cell carcinoma. Sci Rep.

[9] Peinado H (2012). Melanoma exosomes educate bone marrow progenitor cells toward a pro-metastatic phenotype through MET. Nature medicine.

[10] Steinbichler, T. B., Dudas, J., Riechelmann, H. & Skvortsova II. The Role of Exosomes in Cancer Metastasis. *Seminars in cancer biology*, doi:10.1016/j.semcancer.2017.02.006 (2017). 10.1016/j.semcancer.2017.02.006 28215970

[11] Villarroya-Beltri C, Baixauli F, Gutierrez-Vazquez C, Sanchez-Madrid F, Mittelbrunn M (2014). Sorting it out: Regulation of exosome loading. Seminars in cancer biology.

[12] Thomas SN (2013). Exosomal Proteome Profiling: A Potential Multi-Marker Cellular Phenotyping Tool to Characterize Hypoxia-Induced Radiation Resistance in Breast Cancer. Proteomes.

[13] Yentrapalli, R. *et al*. Quantitative changes in the protein and miRNA cargo of plasma exosome-like vesicles after exposure to ionizing radiation. *Int J Radiat Biol*, 1–12, doi:10.1080/09553002.2017.1294772 (2017). 10.1080/09553002.2017.1294772 28264626

[14] Jelonek K (2015). Ionizing radiation affects protein composition of exosomes secreted *in vitro* from head and neck squamous cell carcinoma. Acta biochimica Polonica.

[15] McKelvey KJ, Powell KL, Ashton AW, Morris JM, McCracken SA (2015). Exosomes: mechanisms of uptake. Journal of Circulating Biomarkers.

[16] Segura E, Guérin C, Hogg N, Amigorena S, Théry C (2007). CD8+ Dendritic Cells Use LFA-1 to Capture MHC-Peptide Complexes from Exosomes *In Vivo*. The Journal of Immunology.

[17] Skog J (2008). Glioblastoma microvesicles transport RNA and proteins that promote tumour growth and provide diagnostic biomarkers. Nature cell biology.

[18] Guo BB, Bellingham SA, Hill AF (2016). Stimulating the Release of Exosomes Increases the Intercellular Transfer of Prions. The Journal of biological chemistry.

[19] Ratajczak MZ, Ratajczak J (2016). Horizontal transfer of RNA and proteins between cells by extracellular microvesicles: 14 years later. Clinical and Translational Medicine.

[20] Demory Beckler M (2013). Proteomic analysis of exosomes from mutant KRAS colon cancer cells identifies intercellular transfer of mutant KRAS. Molecular & cellular proteomics: MCP.

[21] Mutschelknaus L (2016). Exosomes Derived from Squamous Head and Neck Cancer Promote Cell Survival after Ionizing Radiation. PloS one.

[22] Knowles JA (2011). Disruption of the AKT pathway inhibits metastasis in an orthotopic model of head and neck squamous cell carcinoma. Laryngoscope.

[23] Smith A, Teknos TN, Pan Q (2013). Epithelial to mesenchymal transition in head and neck squamous cell carcinoma. Oral Oncol.

[24] Sekulic A (2000). A direct linkage between the phosphoinositide 3-kinase-AKT signaling pathway and the mammalian target of rapamycin in mitogen-stimulated and transformed cells. Cancer research.

[25] Zhou H, Huang S (2011). Role of mTOR signaling in tumor cell motility, invasion and metastasis. Curr Protein Pept Sci.

[26] Gao W, Li JZ, Chan JY, Ho WK, Wong TS (2012). mTOR Pathway and mTOR Inhibitors in Head and Neck Cancer. ISRN Otolaryngol.

[27] Coppock JD (2016). mTOR inhibition as an adjuvant therapy in a metastatic model of HPV + HNSCC. Oncotarget.

[28] Magnuson B, Ekim B, Fingar (2012). Diane C. Regulation and function of ribosomal protein S6 kinase (S6K) within mTOR signalling networks. Biochemical Journal.

[29] Park CM (2006). Ionizing radiation enhances matrix metalloproteinase-2 secretion and invasion of glioma cells through Src/epidermal growth factor receptor-mediated p38/Akt and phosphatidylinositol 3-kinase/Akt signaling pathways. Cancer research.

[30] Li X (2013). Overexpression of Bmi-1 contributes to the invasion and metastasis of hepatocellular carcinoma by increasing the expression of matrix metalloproteinase (MMP)2, MMP-9 and vascular endothelial growth factor via the PTEN/PI3K/Akt pathway. Int J Oncol.

[31] Chen JS (2013). Sonic hedgehog signaling pathway induces cell migration and invasion through focal adhesion kinase/AKT signaling-mediated activation of matrix metalloproteinase (MMP)-2 and MMP-9 in liver cancer. Carcinogenesis.

[32] Arscott WT (2013). Ionizing Radiation and Glioblastoma Exosomes: Implications in Tumor Biology and Cell Migration. Translational Oncology.

[33] Bussink J, van der Kogel AJ, Kaanders JH (2008). Activation of the PI3-K/AKT pathway and implications for radioresistance mechanisms in head and neck cancer. Lancet Oncol.

[34] Lui VW (2013). Frequent mutation of the PI3K pathway in head and neck cancer defines predictive biomarkers. Cancer Discov.

[35] Liu Q, Turner KM, Alfred Yung WK, Chen K, Zhang W (2014). Role of AKT signaling in DNA repair and clinical response to cancer therapy. Neuro Oncol.

[36] Cai Y, Dodhia S, Su GH (2017). Dysregulations in the PI3K pathway and targeted therapies for head and neck squamous cell carcinoma. Oncotarget.

[37] Shin SY, Kim CG, Jung YJ, Lim Y, Lee YH (2016). The UPR inducer DPP23 inhibits the metastatic potential of MDA-MB-231 human breast cancer cells by targeting the Akt-IKK-NF-kappaB-MMP-9 axis. Sci Rep.

[38] Iizuka S, Ishimaru N, Kudo Y (2014). Matrix Metalloproteinases: The Gene Expression Signatures of Head and Neck Cancer Progression. Cancers.

[39] Katayama A (2004). Expressions of matrix metalloproteinases in early-stage oral squamous cell carcinoma as predictive indicators for tumor metastases and prognosis. Clinical cancer research: an official journal of the American Association for Cancer Research.

[40] Xie M, Sun Y, Li Y (2004). Expression of matrix metalloproteinases in supraglottic carcinoma and its clinical implication for estimating lymph node metastases. Laryngoscope.

[41] Chien MH, Lin CW, Cheng CW, Wen YC, Yang SF (2013). Matrix metalloproteinase-2 as a target for head and neck cancer therapy. Expert Opin Ther Targets.

[42] de Jong, O. G. *et al*. Cellular stress conditions are reflected in the protein and RNA content of endothelial cell-derived exosomes. *Journal of extracellular vesicles* **1**, doi:10.3402/jev.v1i0.18396 (2012). 10.3402/jev.v1i0.18396 PMC3760650 24009886

[43] Hendrix A (2010). Effect of the secretory small GTPase Rab27B on breast cancer growth, invasion, and metastasis. J Natl Cancer Inst.

[44] McCready J, Sims JD, Chan D, Jay DG (2010). Secretion of extracellular hsp90alpha via exosomes increases cancer cell motility: a role for plasminogen activation. BMC cancer.

[45] Jiao J, Zhao X, Liang Y, Tang D, Pan C (2015). FGF1-FGFR1 axis promotes tongue squamous cell carcinoma (TSCC) metastasis through epithelial-mesenchymal transition (EMT). Biochemical and biophysical research communications.

[46] Leavesley DI (2013). Vitronectin–master controller or micromanager?. IUBMB Life.

[47] Chiu CC (2011). Molecular chaperones as a common set of proteins that regulate the invasion phenotype of head and neck cancer. Clinical cancer research: an official journal of the American Association for Cancer Research.

[48] Koole K (2016). FGFR1 Is a Potential Prognostic Biomarker and Therapeutic Target in Head and Neck Squamous Cell Carcinoma. Clinical cancer research: an official journal of the American Association for Cancer Research.

[49] Gouaze-Andersson V (2016). FGFR1 Induces Glioblastoma Radioresistance through the PLCgamma/Hif1alpha Pathway. Cancer research.

[50] Hofig I (2012). Poloxamer synperonic F108 improves cellular transduction with lentiviral vectors. J Gene Med.

[51] Anastasov, N., Höfig, I., Mall, S., Krackhardt, A. M. & Thirion, C. In *Lentiviral Vectors and Exosomes as Gene and Protein Delivery* Tools (ed. Maurizio Federico) 49–61 (Springer New York, 2016).

[52] Keerthikumar S (2016). ExoCarta: A Web-Based Compendium of Exosomal Cargo. J Mol Biol.

[53] Szklarczyk D (2015). STRING v10: protein-protein interaction networks, integrated over the tree of life. Nucleic acids research.

[54] Wu G, Feng X, Stein L (2010). A human functional protein interaction network and its application to cancer data analysis. Genome Biol.

[55] Shannon P (2003). Cytoscape: a software environment for integrated models of biomolecular interaction networks. Genome research.

