# Supplementary material for: Radiation alters the cargo of exosomes released from squamous head and neck cancer cells to promote migration of recipient cells
Source: Sci Rep. 2017 Sep 29;7:12423. doi: 10.1038/s41598-017-12403-6 (PMC5622080; doi:10.1038/s41598-017-12403-6)
Supplement: Supplementary file 2 — Supplementary Information [file 41598_2017_12403_MOESM2_ESM.pdf]

__Supplementary information__

Radiation alters the cargo of exosomes released from squamous head and neck cancer cells to promote migration of recipient cells

Lisa Mutschelknaus[1], Omid Azimzadeh[1], Theresa Heider[1], Klaudia Winkler[1], Marcus Vetter[2], Rosemarie Kell[1], Soile Tapio[1], Juliane Merl-Pham[3], Stephan M. Huber[4], Lena Edalat[4], Vanja Radulović[1], Nataša Anastasov[1], Michael J. Atkinson[1, 5], Simone Moertl[1*]

[1] Helmholtz Zentrum München, German Research Center for Environmental Health, Institute of Radiation Biology, Neuherberg, Germany.

[2] Independent Scientist, Hofheimerstraße 6, Munich, Germany.

[3] Helmholtz Zentrum München, German Research Center for Environmental Health, Research Unit Protein Science, München, Germany

[4] Department of Radiation Oncology, University of Tübingen, Germany.

[5] Chair of Radiation Biology, Technical University of Munich, Munich, Germany.

[*]Corresponding author: Simone Moertl

E-Mail: moertl@helmholtz-muenchen.de

Phone number: +49 89 3187-3143

Fax number: +49 89 3187-3378

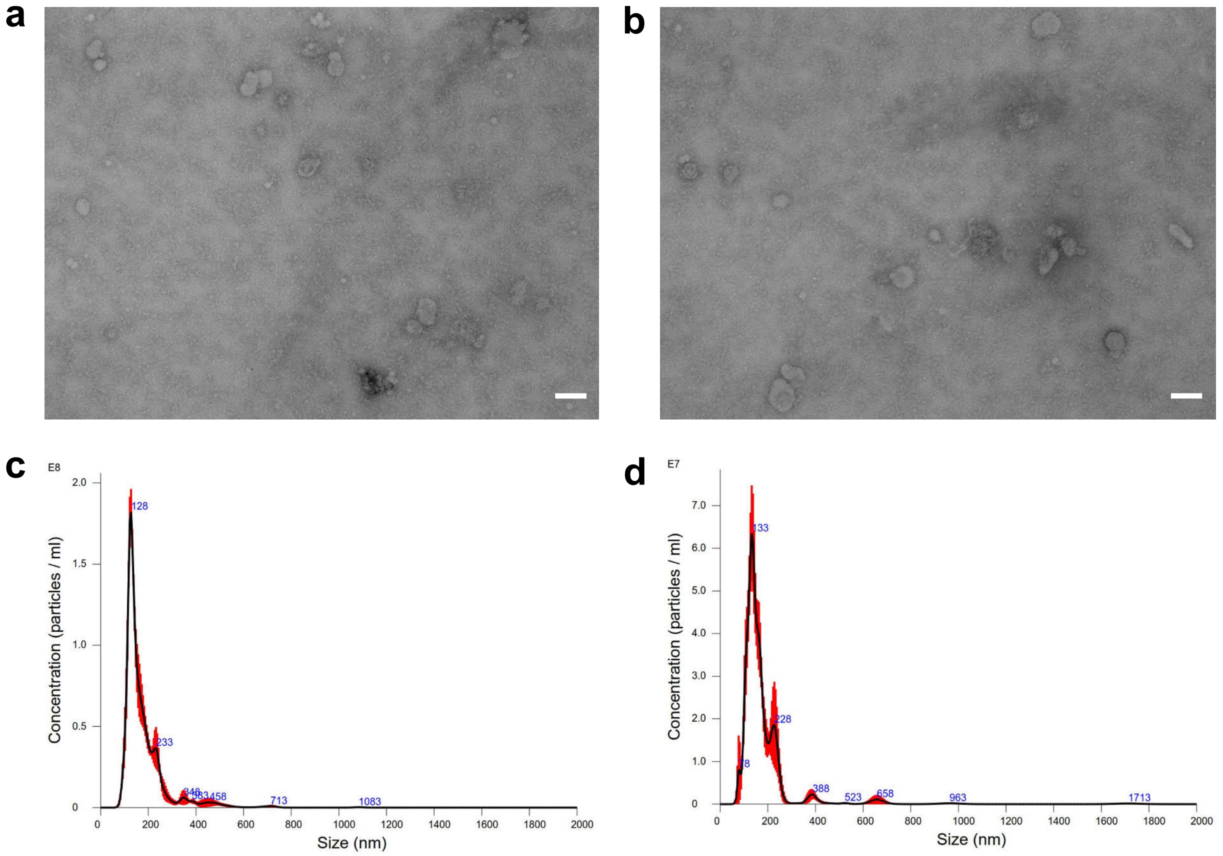

Supplementary Figure S1. Characterization of isolated HNSCC exosomes. (a) Electron microscopy of BHY (EXO 0 Gy) exosomes (scale bar: 100 nm). **(b)** Electron microscopy of FaDu (EXO 0 Gy) exosomes (scale bar: 100 nm). **(c)** Size distribution of BHY (EXO 0 Gy) exosomes measured with NanoSight technology. **(d)** Size distribution of FaDu (EXO 0 Gy) exosomes measured with NanoSight technology.

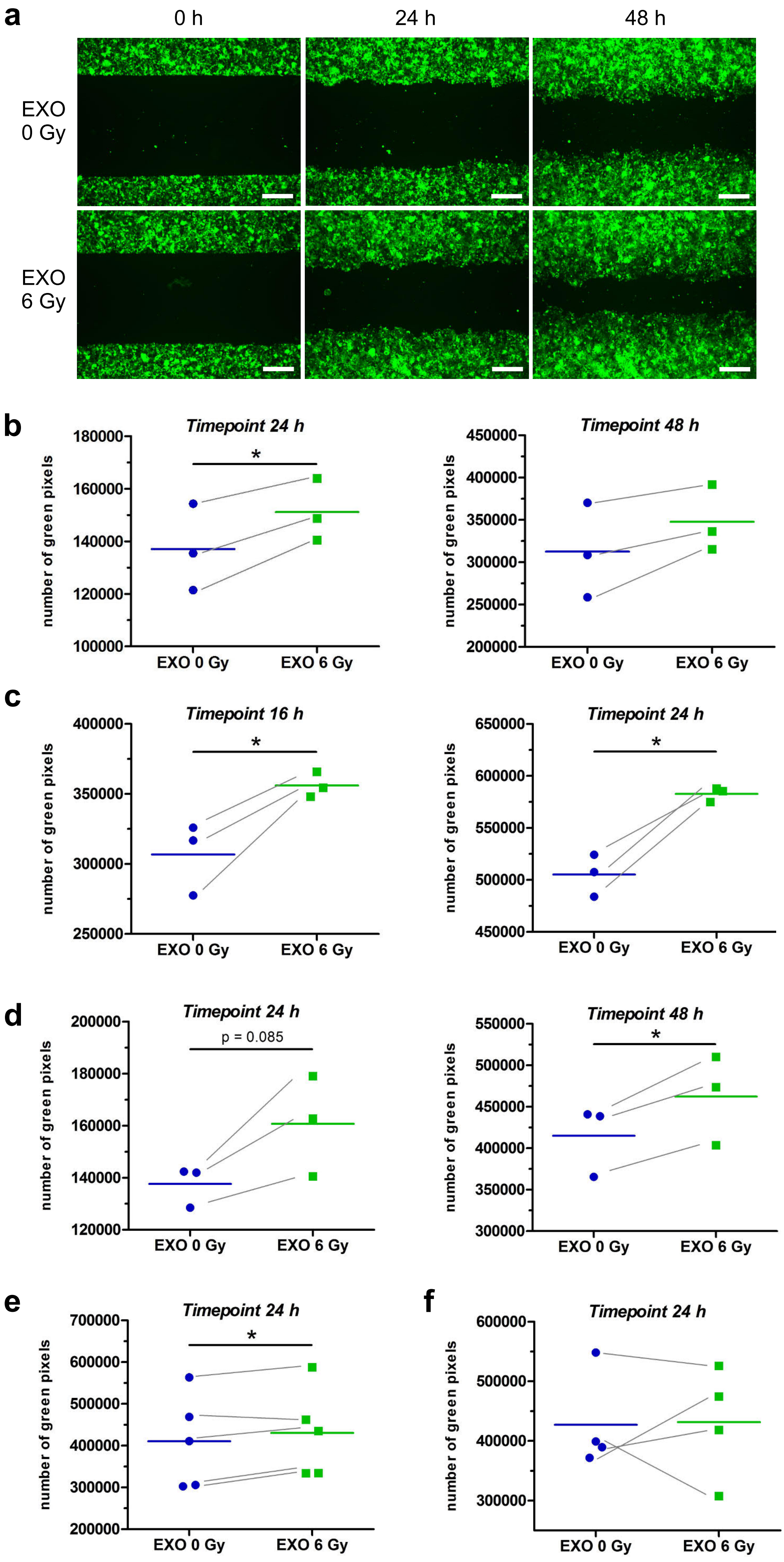

Supplementary Figure S2. Effect of exosomes isolated after irradiation of tumour and non-tumour cell lines on HNSCC migration. (a, b) Exemplary wound healing of FaDu-GFP cells after 24 and 48 hours (scale bar: 500 µm). Cells were either preincubated with exosomes from non-irradiated (EXO 0 Gy; upper row) or from 6 Gy irradiated FaDu cells (EXO 6 Gy; lower row). **(c)** BHY-GFP cells were either preincubated with exosomes from non-irradiated (EXO 0 Gy) or 6 Gy (EXO 6 Gy) irradiated FaDu cells. **(d)** FaDu-GFP cells were either preincubated with exosomes from non-irradiated (EXO 0 Gy) or 6 Gy (EXO 6 Gy) irradiated BHY cells. **(e)** BHY-GFP cells were either preincubated with exosomes from non-irradiated (EXO 0 Gy) or 6 Gy (EXO 6 Gy) irradiated IBR3 cells. **(f)** BHY-GFP cells were either preincubated with exosomes from non-irradiated (EXO 0 Gy) or 6 Gy (EXO 6 Gy) irradiated HCAEC cells. Quantification of the wound healing capacity with the Image Colour Analyser after 16, 24 and 48 hours [$n \geq 3$; two-sided, paired t-test; p-value < 0.05].

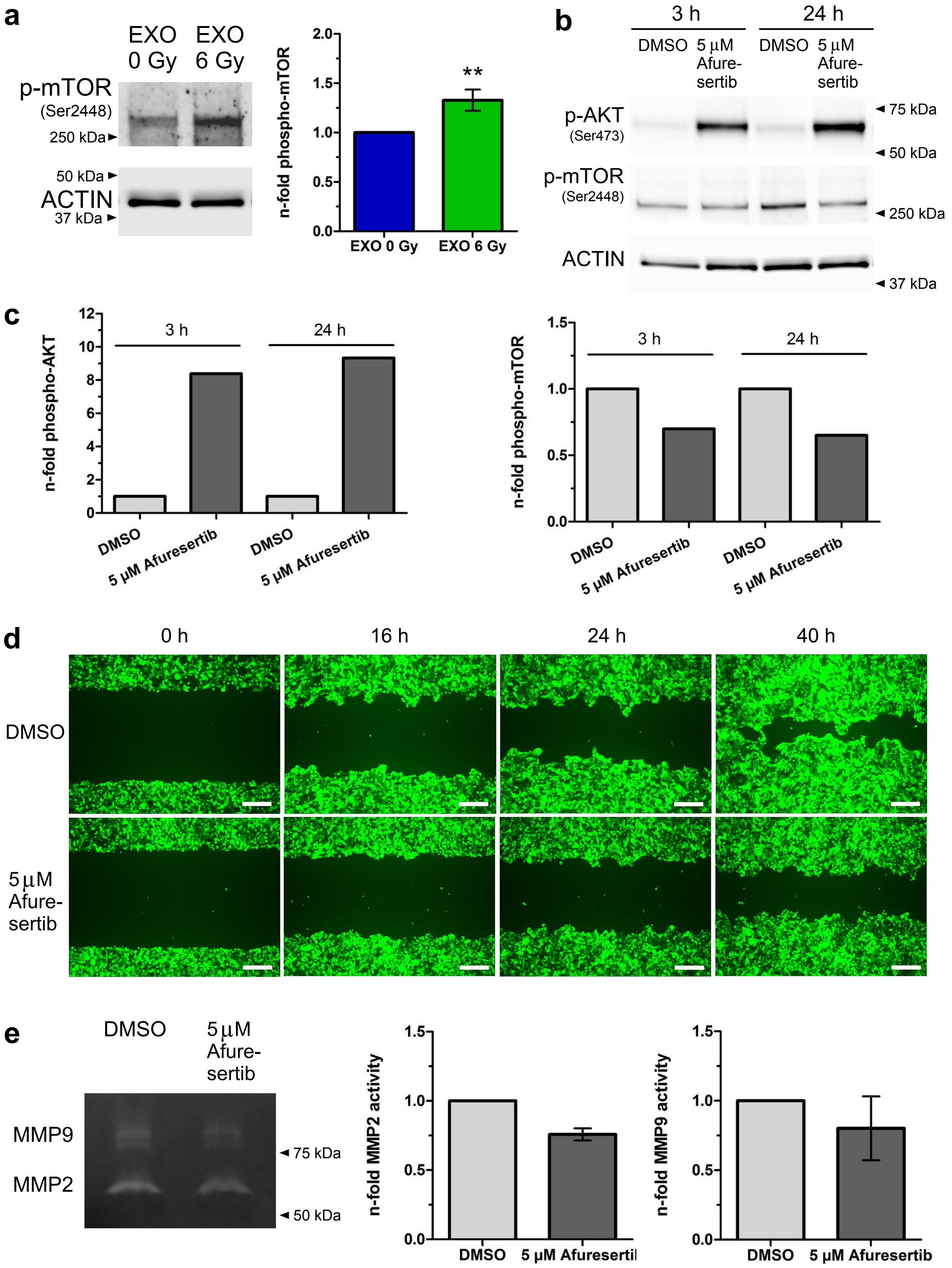

Supplementary Figure S3. Effect of the AKT-inhibitor Afuresertib on molecular targets and migration. (a) Western blot of phospho-mTOR (Ser2448) of cells which were incubated for 3 hours with exosomes isolated either from irradiated cells (EXO 6 Gy) or from non-irradiated cells (EXO 0 Gy). Normalization was performed to ACTIN and to cells treated with exosomes from non-irradiated cells (EXO 0 Gy). Cropped blots are displayed [n = 4; ±SD; two-sided, one-sample t-test; p-value < 0.01]. **(b, c)** Western blot of phospho-AKT (Ser473) and phospho-mTOR (Ser2448) of cells which were treated with 5 µM Afuresertib or DMSO for 3 or 24 hours. Normalization was performed to ACTIN and to control cells treated with DMSO. Cropped blots are displayed [n = 1]. **(d)** Exemplary wound healing of BHY-GFP cells after treatment with 5 µM of the AKT-inhibitor Afuresertib, or with the equal volume of DMSO. The pictures were taken 16, 24 and 40 hours after migration start (scale bar: 500 µm). **(e)** MMP2 and MMP9 activity in the supernatants 24 hours after treatment of BHY cells with 5 µM Afuresertib or DMSO. Normalization was performed to control cells treated with DMSO. Cropped gels are displayed [n = 2; ±SD].

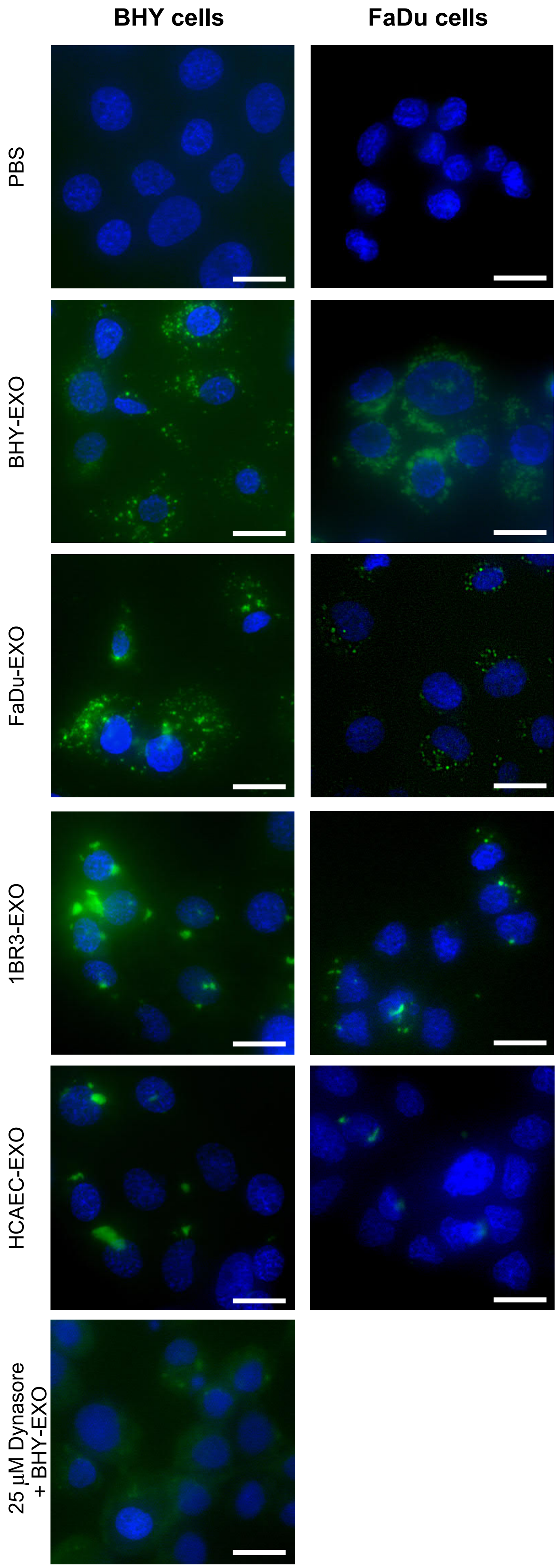

Supplementary Figure S4. Exosomes from tumour and non-tumour cells transfer proteins to recipient BHY and FaDu cells. Exosomal proteins of BHY (EXO-BHY), FaDu (EXO-FaDu), IBR3 (EXO-IBR3), HCAEC (EXO-HCAEC) cells and PBS as negative control were stained with CFSE and subsequently transferred onto recipient BHY and FaDu cells. In addition BHY cells were pretreated with 25 µM Dynasore for 1 hour with subsequent transfer of BHY exosomes (25 µM Dynasore + BHY-EXO). The protein uptake was monitored after 24 hours of exosome exposure (scale bar: 25 µm).

Process analysis with String

Network: Deregulated proteins

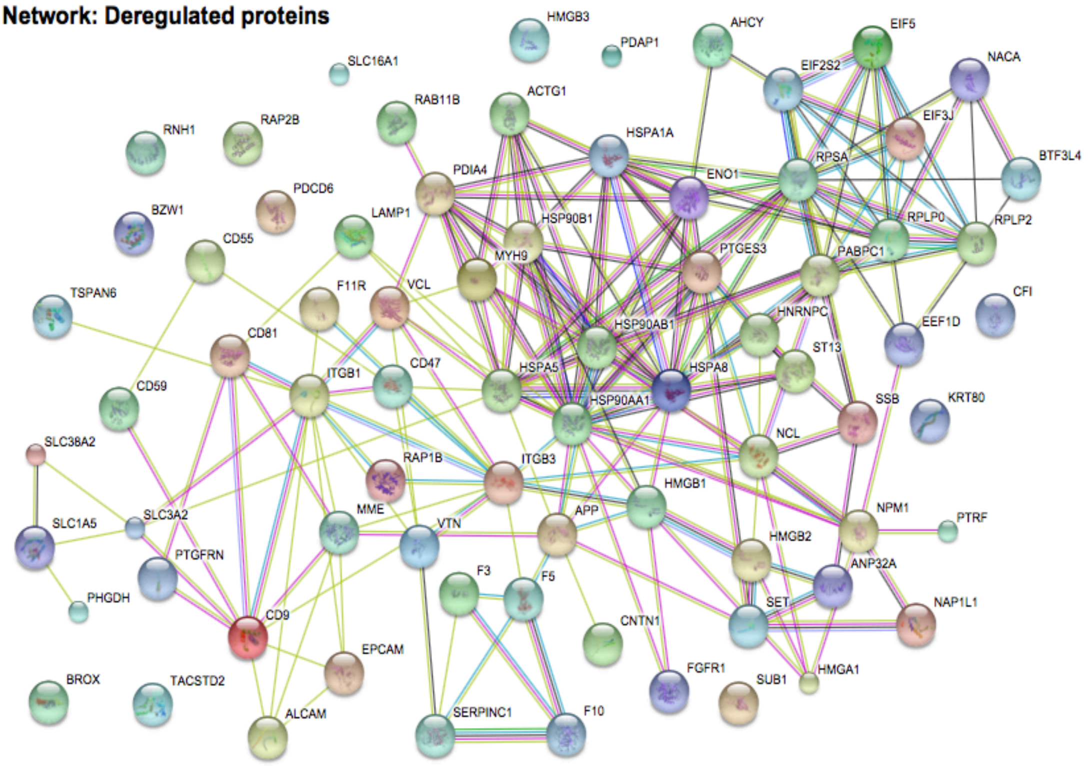

Edge legend

- from curated databases } Known interactions
- experimentally determined } Known interactions
- gene neighborhood } Predicted interactions
- gene co-occurence } Predicted interactions
- textmining
- co-expression
- protein homology

Network Stats

number of nodes	75
number of edges	196
average node degree	5.23
avg. local clustering coefficient	0.474
expected number of edges	77
PPI enrichment p-value	0

Supplementary Figure S5. Interaction network of radiation-regulated exosomal proteins. *In silico* STRING analysis of deregulated exosomal proteins to determine protein interaction. The minimum required interaction score was set to medium confidence (0.004) and the analysis revealed a high degree of interaction indicated by the PPI enrichment p-value $< 1 \times 10^{-15}$.

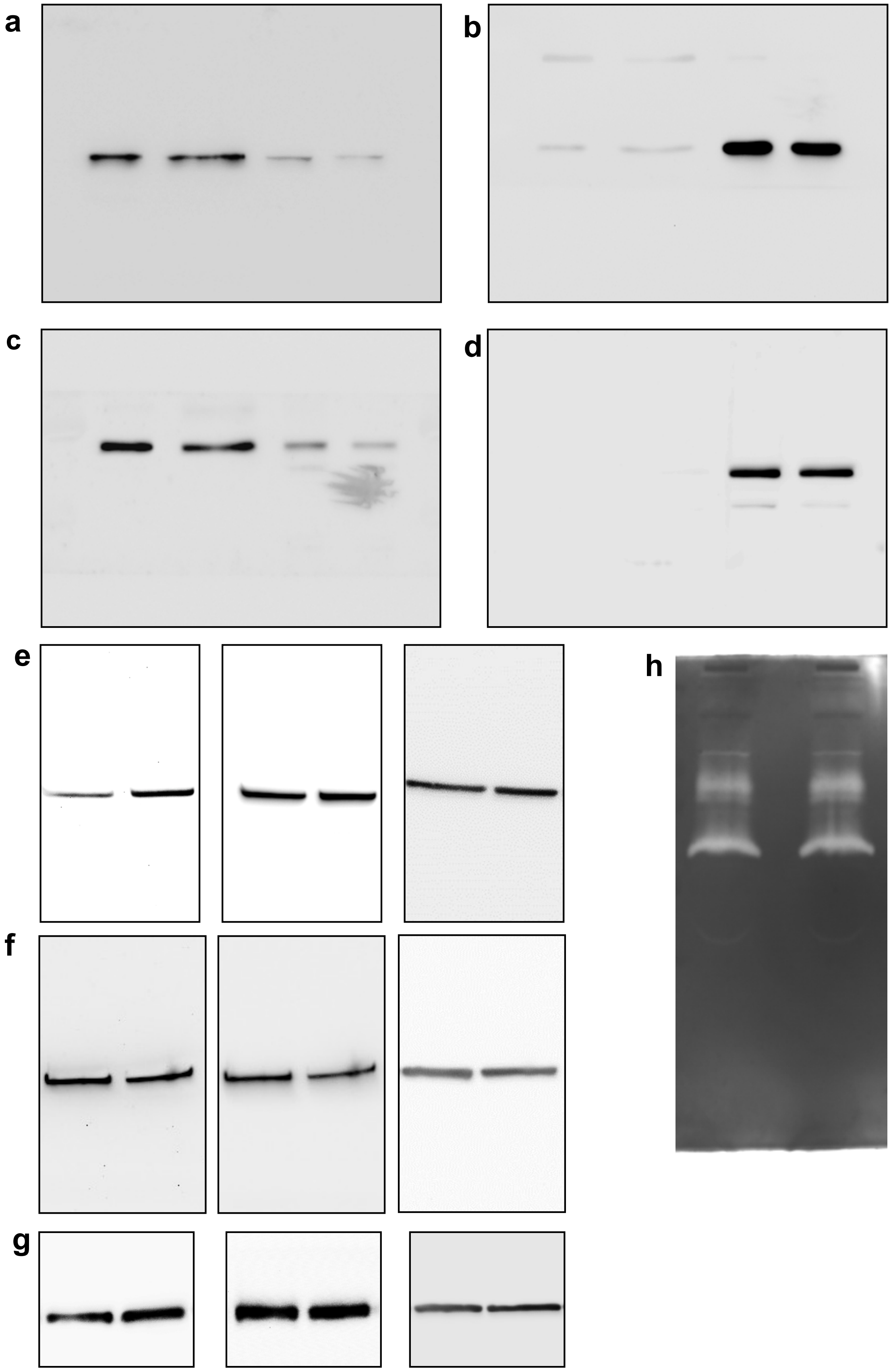

Supplementary Figure S6. Full-length western blots and gels. (a) Western blot Alix (Fig 1 b) **(b)** Western blot GAPDH (Fig 1 b) **(c)** Western blot TSG101 (Fig 1 b) **(d)** Western blot Calnexin (Fig 1 b) **(e)** Western blot phospho-mTOR (Ser2448), mTOR and Actin (Fig 4 a) **(f)** Western blot phospho-mTOR (Ser2448), mTOR and Actin (Fig 4 b) **(g)** Western blot phospho-rpS6 (Ser240/244), rpS6 and Actin (Fig 4 c) **(h)** Zymography (Fig 4 d).
